# Epigenetic Control of Macrophage Polarisation and Soluble Mediator Gene Expression during Inflammation

**DOI:** 10.1155/2016/6591703

**Published:** 2016-04-10

**Authors:** Theodore S. Kapellos, Asif J. Iqbal

**Affiliations:** Sir William Dunn school of Pathology, South Parks Road, Oxford OX1 3RE, UK

## Abstract

Macrophages function as sentinel cells, which constantly monitor the host environment for infection or injury. Macrophages have been shown to exhibit a spectrum of activated phenotypes, which can often be categorised under the M1/M2 paradigm. M1 macrophages secrete proinflammatory cytokines and chemokines, such as TNF-*α*, IL-6, IL-12, CCL4, and CXCL10, and induce phagocytosis and oxidative dependent killing mechanisms. In contrast, M2 macrophages support wound healing and resolution of inflammation. In the past decade, interest has grown in understanding the mechanisms involved in regulating macrophage activation. In particular, epigenetic control of M1 or M2 activation states has been shown to rely on posttranslational modifications of histone proteins adjacent to inflammatory-related genes. Changes in methylation and acetylation of histones by methyltransferases, demethylases, acetyltransferases, and deacetylases can all impact how macrophage phenotypes are generated. In this review, we summarise the latest advances in the field of epigenetic regulation of macrophage polarisation to M1 or M2 states, with particular focus on the cytokine and chemokine profiles associated with these phenotypes.

## 1. Macrophages Are a Heterogeneous Population Tightly Controlled by Tissue-Specific Factors

Macrophages are immune cells of myeloid lineage that originate from the embryonic yolk sac and are an integral component of the host's immune response. They act as sentinel cells, which constantly sample their microenvironment and their primary function is to monitor tissues for potential threats (e.g., infection and injury). There are a range of resident macrophage populations, including microglia (brain), Kupffer cells (liver), alveolar macrophages (lungs), splenic macrophages, osteoclasts, bone marrow macrophages, histiocytes (connective tissue), intraocular macrophages (eye), subcapsular sinusoidal macrophages (lymph nodes), and intestinal macrophages [1, 2].

All macrophage populations monitor their tissue of residence and respond to pathogen-associated molecular patterns (PAMPs) and danger-associated molecular patterns (DAMPs) by initiating the acute inflammatory cascade. After clearance of the pathogenic factors, resident macrophages replenish their populations by proliferation and promote the resolution of inflammation by clearing apoptotic cells/debris and support wound healing and tissue repair programs [1, 3].

In addition to these generic roles, macrophages demonstrate tissue-specific functional properties [3]. For example, resident populations in mucosal areas come into contact with environmental pathogens and splenic macrophages control iron metabolism together with Kupffer cells and clear senescent erythrocytes from the circulation. In the brain, microglia cells play a crucial role in neuronal survival [3].

To carry out their tissue-specific functions, macrophages respond to local signals released in their niche. For instance, it has been shown that retinoic acid in the peritoneal cavity and TGF-*β*, CSF-1, and IL-34 in the brain help define peritoneal macrophages and microglia populations, respectively [2, 4]. CSF-1 and RANKL induce differentiation of osteoclasts, CSF-2 secretion by the respiratory epithelium matures alveolar macrophages, and IL-10 prevents excessive intestinal macrophages activation [2, 4]. Under this constant conditioning, the macrophage lineage is established via expression of signature transcription factors (TFs), which dictate their functions not only in homeostasis, but also during an immune response.

Examples include the ability of peritoneal macrophages to signal via eicosanoid products, microglia to undergo oxidative metabolism, alveolar macrophages to metabolise lipids, and red pulp splenic macrophages to respond to interferon activation [5]. Recently, many reports have identified core genes that define macrophage populations in the host. Tissue-specific TF regulate tissue signature genes by binding to motifs on enhancers to control gene expression. For example,* Spic* has been shown to be the signature TF for red pulp splenic macrophage development,* Gata6* is responsible for* Tgfb2*,* Cebpb*, and* Rarb* expression in peritoneal macrophages,* Runx3* in intestinal macrophages,* Car4* in alveolar macrophages,* Clec4f* in Kupffer cells, and* Mef2c* in microglia [2, 4–7]. In some cases, subpopulations of resident macrophages may express their own selective signature genes; for instance, small peritoneal macrophages express* Ciita*, which is detected at low levels in large peritoneal macrophages [4].

These transcriptional differences result in selective expression of proteins by different macrophage populations, for instance, VCAM-1 and CD31 in splenic macrophages, CX3CR1 and Siglec-H in microglia, ICAM-2 and CD93 in peritoneal macrophages, CCR2 in monocytes, CLEC4F in Kupffer cells, or CD11a and EpCAM in alveolar macrophages [2, 5, 6]. These proteins are critical in recruiting and integrating macrophage populations into their respective niches.

## 2. Macrophage Differentiation and Immune Responses Are Regulated by Complex Epigenetic Changes

Macrophages are constantly sampling their ever changing environment and have therefore evolved regulatory epigenetic programs that define their core functions and also allow them to respond to environmental cues rapidly.

**Figure 1:**
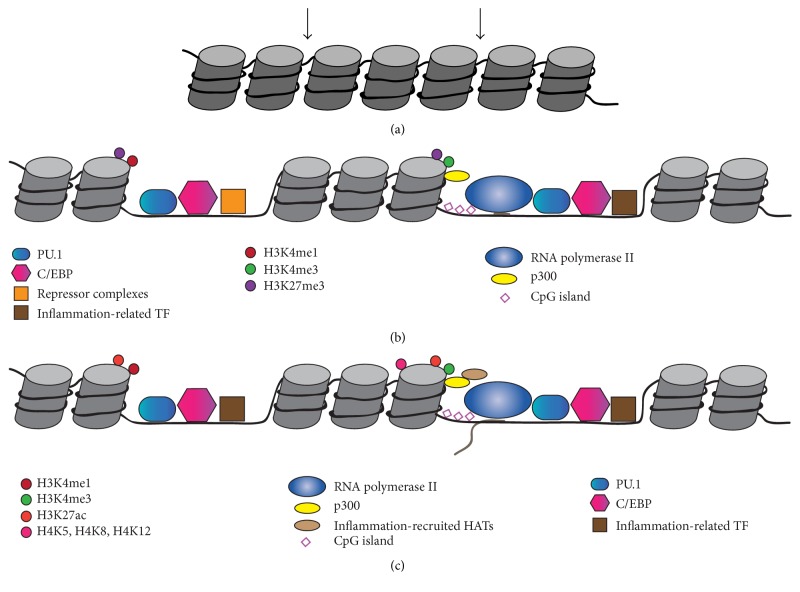
Epigenetic regulation in macrophages in homeostasis and inflammation. (a) During lineage establishment, the master macrophage regulator PU.1 unpacks the tight organisation of chromatin and binds to its motifs on the DNA sequence (arrows). Additional macrophage-restricted TFs interact with PU.1 and are subsequently recruited to the loosened DNA resulting in establishment of nucleosome-free regions at macrophage enhancers and promoters. (b) Enhancers are epigenetically marked by the H3K4me1 signature, whereas promoters are H3K4me3 labelled. Genes which are not active at baseline may be poised, meaning that their enhancers are marked by H3K27me3 signatures, rendering them ready to promote gene transcription in the presence of an appropriate stimulus. One category of such genes is the primary response genes, which exhibit active repression at their enhancers and are transcribed at low levels. (c) In the presence of local signals, these genes lose the suppressive H3K27me3 mark on their enhancers and promoters and are acetylated at H3K27 by the constitutively present p300 and recruited acetyltransferases. The produced transcripts are successfully elongated and leave the nucleus for protein synthesis. This conversion is facilitated by inflammation-related TF, which bring enhancers close to gene promoters to initiate gene transcription.

The lineage determination of macrophages is accomplished by the constitutive expression of the ETS-domain TF and PU.1. PU.1 can bind to its motifs on DNA and facilitate stable opening of chromatin and recruitment of additional TF (Figures 1(a) and 1(b)). It is found at macrophage gene enhancers and promoters and contributes to enhancer organisation [8–10].* In vitro* ectopic expression of PU.1 in fibroblast cell lines has been shown to induce the expression of macrophage-specific genes, such as* Cd68*,* Emr1*, and* Lyz2*, illustrating its significance for the establishment of the macrophage lineage [11].

However, recent evidence has shown that PU.1 does not establish the macrophage lineage on its own (Figure 1(b)). In fact, PU.1 binding motifs on promoters are in close proximity to other macrophage TF, such as AP-1, C/EBP*α*, C/EBP*β*, and RUNX. Evidence from Heinz et al. suggests that cross-talk between PU.1 and C/EBP*β* supports the organisation of the macrophage enhancer landscape [8].

The basic unit of DNA organisation in cells, the nucleosome, consists of 147 base pairs of DNA wrapped around a dimer of tetramers of the core histone proteins, H2A, H2B, H3, and H4. The nucleosomes pack the DNA efficiently in the nucleus, but at the same time they obstruct DNA from being transcribed. In order for transcription to occur, the core histones undergo modifications and unravel in a process called nucleosome remodelling. Key histone modifications that regulate gene expression include H3 and H4 methylation and acetylation [12].

During macrophage differentiation, lineage-specific enhancers and promoters are marked epigenetically with histone modifications [10]. Enzymes transfer methyl groups to histone tails resulting in positive or negative regulation of adjacent gene expression. The chemical reaction is targeted to lysine (K) or arginine (R) residues on H3 and H4 and the enzymes show high selectivity for their targets [13, 14]. Gene promoters are labelled with H3K4me3, whilst enhancers are marked with H3K4me1 (Figure 1(b)). Promoters are invariably labelled with H3K4me3, regardless of whether the genes are expressed or not. They may acquire additional H3K9/14ac marks as a signature of transcription initiation and H3K36me3 and H3K79me2 as signatures of transcription elongation [15].

Regulation of gene expression in macrophages has been reported to occur mainly at the level of enhancers. Active enhancers present deposition of H3K27ac [16] and therefore feature as H3K4me1^hi^H3K27ac^hi^ and H3K4me3^hi^H3K27ac^hi^, respectively [17]. In contrast, repressive marks on enhancers, such as H3K27me3, indicate a poised status of activation, meaning that particular enhancer has the potential to be activated [17].

The combination of cell-specific TF and chromatin modifications shapes the epigenetic landscape of macrophages, thereby defining the spectrum of responses that these cells are capable of carrying out [17]. Each cell type possesses a unique enhancer landscape that allows them to carry out cell-specific functions [18]. To further illustrate the notion that cell functions are dependent on preestablished epigenetic patterns, Creyghton et al. showed that nuclear reprogramming of fibroblasts into fibroblast-derived induced pluripotent stem cells resets the global enhancer patterns to embryonic stem (ES) cell configuration [16]. Reciprocally, gains and losses of H3K27ac marks on genes expressed by differentiated neural cells were observed in comparison with ES cells and neural progenitors, which suggests that cell types alter their epigenetic landscape during differentiation [16].

After the establishment of the macrophage lineage by pioneer TF, some genes are additionally bound by second-tier TF, also referred to as “primers” [19]. Exemplified by Atf3, JunB, and AP-1, primer TF marks the potential of the cells to respond to environmental stimuli, and it is believed that they attract inflammation-related TF upon stimulation [19]. When macrophages are primed by an environmental cue, they need to respond rapidly and therefore acute chromatin remodelling is required (Figure 1(c)). PU.1 in combination with C/EBP is responsible for the induction of many LPS- and TNF-responsive genes [8, 20]. In addition, p300 and other acetyltransferases [21, 22] are recruited to H3K4me1^hi^ enhancers, enriched at LPS-induced genes, where they acetylate H3 and H4 histones [11, 22]. The recruitment of inflammation-related TF to enhancers, such as NF-kB, IRF proteins, AP-1, and FOS, suggests that p300, together with the constitutively active PU.1 and recruited TF, regulates gene expression and immune responses of these myeloid cells [11]. For example, in IFN-*γ*-stimulated macrophages, STAT1 is recruited to selected H3K4me1^hi^ enhancers and induces the expression of IFN-*γ* responsive genes [18].

Gene activation kinetics upon TLR4 ligation revealed that the induced genes fall into two groups; the primary response genes, induced independently of new protein synthesis shortly after macrophage activation, and the secondary response genes, the expression of which is elevated many hours after ligation [23, 24]. Although both groups of genes have similar H3K4me3 and H3K9/14ac distribution on their promoters under basal conditions, the former exhibits increased RNA polymerase occupancy at their promoters and low levels of gene transcription [9, 25, 26]. In addition, the promoters of primary response genes are enriched for NF-kB, AP-1/CREB, and SRF factor motifs, whereas the secondary response genes possess interferon-sensitive response elements (ISREs) binding sites [9].

Elegant work by Ramirez-Carrozzi et al. provided an insight into the mechanistic framework of primary and secondary response gene expression in macrophages during LPS activation [27]. High CpG content in promoters of early primary response genes correlates with loosened conformation of chromatin. In contrast, genes that lack CpG island-rich promoters require further nucleosome remodelling by the SWI-SNF nucleosome remodeller in order to be expressed. Following LPS stimulation, H4K5, H4K8, and H4K12 are acetylated by p300 and the recruited acetyltransferases GCN5 and PCAF at both primary and secondary response gene promoters [25].

Recently, Ostuni et al. discovered another subset of enhancers termed latent enhancers, which are neither PU.1 nor H3K4me1 marked under basal conditions but are induced upon macrophage activation [28]. Latent enhancers induce late-transcribed genes and the TF they depend on for their expression varies according to the environmental stimulus responsible. Interestingly, the authors showed that removal of IFN-*γ* and IL-4 retained the induced H3K4me1 marks on the enhancers and preserved these elements in poised conformation [28].

The existence of epigenetic regulation of macrophages is not only essential for the induction of their activation and participation in the inflammatory process, but is also required for the inhibition of the immune response and avoidance of excessive inflammation and tissue damage [23]. Corepressor complexes, such as the NCoR, SMRT, CoREST, mSin3A, and RCoR or the CTCF factor, are recruited to gene promoters under the basal state and need to be displaced by coactivators of gene expression [21, 25]. Bcl-6 is a TF that controls expression of more than one-third of all LPS-responsive genes. It acts to antagonise NF-kB binding at enhancers and is essential for transcriptional repression [29]. During LPS stimulation, macrophage gene expression is tightly controlled by inducible signal- and gene-specific regulators, which aim to suppress gene expression at either the posttranscriptional or the posttranslational level [21].

The remainder of this review will discuss the epigenetic changes that occur upon activation of macrophages and influence polarisation to the M1 or the M2 phenotype. We focus particularly on the role of four broad families of enzymes that are responsible for altering the condensation of chromatin during inflammatory conditions, resulting in gene induction or suppression and how these enzymes interfere with the methylation and acetylation motifs at the promoters of genes to enhance or inhibit their transcription and dictate the overall responses of macrophages to various environmental stimuli.

## 3. Activated Macrophages Acquire Different Polarisation States

Macrophages are activated and respond to mount an effective immune response against potentially harmful agents, such as PAMPs, DAMPs, or tumours. The outcome of macrophage activation depends on the inflammatory stimulus. Historically, macrophage activation states have been summarised under the “M1/M2 paradigm.” The M1/M2 paradigm comes as a reflection of the T_H_1/T_H_2 paradigm of T helper cell activation and embodies the activation status of macrophages primed with IFN-*γ*, LPS, viral products, or GM-CSF (M1 macrophages or classically activated) or IL-4, IL-10, glucocorticoids, or M-CSF (M2 macrophages or alternatively activated). In many studies, M2 macrophages have been further divided into M2a (IL-4 induced), M2b (IgG induced), and M2c (IL-10 and glucocorticoid induced) despite the fact that a consensus has been reached to instead define macrophage phenotypes based on the activator used [81].

Induction of the M1 or M2 phenotype in macrophages is associated with a complex network of signalling intermediate effectors and TF. JNK, PI3K/Akt, Notch-Jagged, and cytokine-induced JAK/STAT pathways have all been implicated in skewing macrophage responses to one state or the other, leading to TF-mediated gene expression [82, 83].

Recently, microRNAs have been reported to play a pivotal role in macrophage polarisation and much attention has since focused on their indirect contribution to immunopathologies (reviewed in [83, 84]). Some microRNAs have been associated with M1 macrophage functions, such as miR-29b, miR-125-a-5p, and miR-155 [85], whereas others, such as miR-21, miR-146a, miR-155, miR-124, miR-223, and let-7c, have been linked to the M2 macrophage anti-inflammatory properties [86–92].

M1 activated macrophages acquire a proinflammatory phenotype and secrete high levels of IL-12 and IL-23 and T cell recruiting chemokines, such as CXCL9 and CXCL10, but low levels of IL-10. M2 activated macrophages secrete CCL17, CCL22, and CCL24 and IL-10 and express IL-1ra on their surface [93]. M1 macrophages are poised to kill intracellular pathogens and promote T_H_1 responses, whereas M2 macrophages clear parasitic infections and promote tissue remodelling. The two activation states can be characterised by certain markers. For example,* Nos2* and* Il12* are referred to as M1 activation markers, with* Arg1*,* Ym1*,* Fizz1*, and* Mgl* assigned to the M2 class.

It is important to keep in mind that these polarisation states are not stable* in vivo*; macrophages display a high degree of plasticity, and activation states can often coexist or change during disease progression upon exposure to microenvironment nascent mediator release [81]. In support of this, human macrophages primed with a range of activation stimuli acquired a spectrum of activated phenotypes ranging from the classical to the alternative pathways with shared and specific transcriptional signatures [94].

The presence of polarised macrophages has been linked to pathologies in animal models [95]. Resembling the T_H_1-T_H_2 paradigm, M1 macrophages have been associated with antitumour activity [96], whereas M2 polarised macrophages have been described in models of asthma and parasite infection [97, 98]. Interestingly, adipose tissue macrophages from lean mice have been reported to express M2 signature genes [99], whereas high fat diet induces the recruitment of bone marrow-derived macrophages, which express M1 markers and contribute to the pathology in the adipose tissue [100].

It is clear that, in order to pharmacologically intervene in diseases where macrophages play a fundamental role, there is a need to understand the mechanisms by which these cells acquire new phenotypes* in vitro* and* in vivo*.

## 4. Histone Methyltransferases (HMTs)

The domain that primarily catalyses lysine methylation is called Suppressor of variegation-Enhancer of zeste-Trithorax (SET) and additional protein sequences define HMTs into eight distinct subfamilies. KMT2, KMT3, and KMT7 subfamily members leave positive marks on H3K4 and H3K36, whereas KMT1, KMT5, KMT6, and KMT8 leave repressive marks on H3K9, H4K20, and H3K27. KMT4 is the only HMTs subfamily with no SET domain and is responsible for H3K79 methylation. SET7/9 can additionally methylate nonhistone proteins, such as p53, NF-kB, DNA methyltransferase 1, and others [14].

Changes in histone conformations have been extensively reported to occur during priming of macrophages with LPS, IL-4, and IFN-*γ* [101]. Epigenetic effects on histones cause the formation of DNA loops that bring together distant chromatin sequences and regulate transcription. Innate (e.g., MARCO, CD200, and CD200R1), classical (e.g., H2-Eb1), and alternative (e.g., MRC1) activation markers are some of the genes readily affected resulting in macrophage polarisation [101].

**Figure 2:**
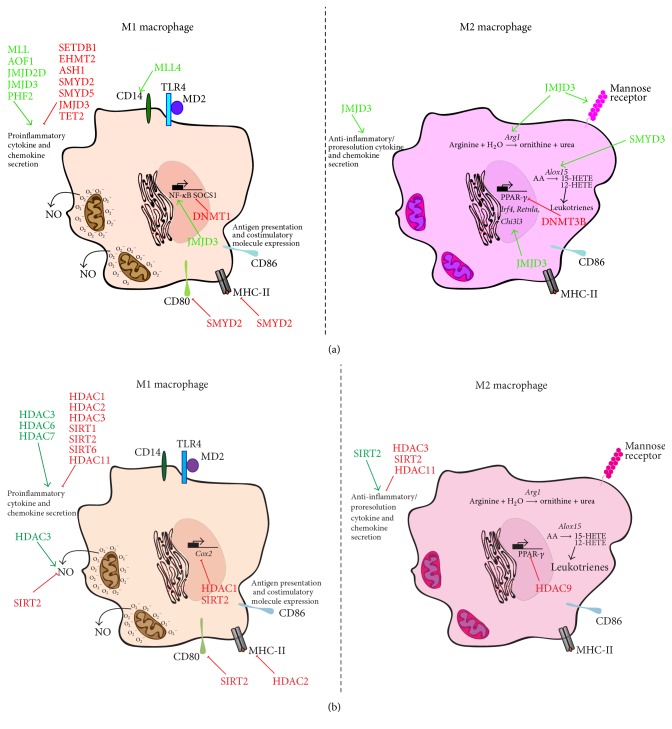
Histone methylation and acetylation status affect gene expression and macrophage polarisation to the M1 or the M2 phenotype. (a) HMTs induce the secretion of proinflammatory cytokines and chemokines in the cell microenvironment and stabilise the levels of CD14 on the macrophage surface. In contrast, some HMTs suppress the expression of MHC-II and costimulatory molecules and modulate the secretion of proinflammatory mediators. These enzymes contribute significantly to the M2 state, inducing the expression of M2 signature markers and the secretion of anti-inflammatory cytokines. (b) Although histone acetylation by HATs is generically associated with gene expression initiation, HDACs can skew the phenotype of macrophages equally to the M1 or the M2 phenotype. Depending on the HDACs subfamily, these enzymes have been shown to affect proinflammatory/proresolution cytokine secretion, MHC-II and costimulatory molecule expression, secretion of ROS and NO, and control of polarisation-determining TF, arachidonic acid (AA), and 12-hydroxyeicosatetraenoic acid (12-HETE).

Macrophage polarisation is differentially regulated by different KMT subfamilies and in some cases by different members within one subfamily (Figure 2(a)). HMTs may switch on the expression of genes, such as cytokine and NO expression [30], or suppress gene expression by methylating negative histone tails. In general terms, HMTs promote polarisation of macrophages towards the M2 phenotype (Table 1). For instance, trimethylation of H3K9 by the KMT1 member SETDB1 silences* TNF* transcription [31], whereas dimethylation of H3K9 results in* Ifn* and downstream interferon-stimulated gene (ISG) suppression in dendritic cells (DCs) and macrophages [32].

The KMT2 members are among the most well-studied HMTs and are associated with M1 macrophage polarisation. The expression of MLL is enhanced in M1 polarised human macrophages and is responsible for H3K4 trimethylation of signature gene promoters, such as* CXCL10* [34]. In contrast, other KMT2 members induce the transcription of inflammation inhibitory genes, most likely to modulate or even terminate macrophage responses. For example, Ash1 trimethylates the* Tnfaip3* promoter to induce the expression of the TLR-antagonising protein A20, which then suppresses IL-6 secretion by peritoneal macrophages [36].

HMTs can also interfere with upstream macrophage activation signalling. Genetic deletion of MLL4 is linked to impaired CD14 surface expression on LPS-stimulated macrophages. Austenaa et al. demonstrated that* Pigp*, an essential component of the GPI-GlcNAc transferase, is one of several hypomethylated genes following MLL4 ablation in macrophages, leading to defective CD14 GPI-anchoring on the cell surface [35].

KMT3 enzymes have been reported to contribute to M2 polarisation. LPS-stimulated macrophages downregulate SMYD2 to prevent the H3K36 dimethylation-mediated repression of* Tnf*,* Il6*, MHC-II, and CD40/80 expression [37]. Another member of this family, SMYD3, is overexpressed in M2 polarised macrophages and is responsible for alternative activation epigenetic remodelling, such as H3K4 trimethylation of* ALOX15* [34]. Acting on a different lysine residue, SMYD5 reversibly trimethylates H4K20 to shut down transcription of LPS-induced genes, such as* Tnf* and* Cxcl10* [38].

## 5. DNA Methyltransferases (DNMTs)

In addition to histone methylation, DNMT enzymes carry out DNA cytosine methylation and are divided into four distinct families: DNMT1, DNMT2, DNMT3 (consisting of DNMT3a, DNMT3b, and DNMT3L), and the chromomethylase family, which is exclusive to plants [102].

DNA methylation occurs in intragenic, intergenic, and CpG islands in promoter regions in mammals [103]. Methylation of promoters leads to gene expression silencing, whilst methylation of intragenic regions can induce the expression of alternative transcripts which are tissue- and cell-specific [103]. The presence of numerous hypomethylated regions in intragenic and intergenic regions in macrophages is associated with gene expression in these cells, underlining the influence of global methylation on gene expression [104].

DNA methylation of CpG islands in gene promoters has been shown to shift macrophages towards both M1 and M2 phenotypes by inactivating state-specific signature genes. Methylation of CpG islands impacts negatively on the expression of genes. For example, DNMT1 hypermethylates SOCS1 gene promoter during LPS activation of macrophages and prolongs the secretion of proinflammatory mediators, such as TNF-*α* and IL-6 [40]. Hypermethylation of gene promoters by DNMT1 and DNMT3b exacerbates the outcome in an experimental model of atherosclerosis by repressing the expression of cystathionine-*γ*-lyase [41].

Genome-wide methylated DNA sequencing of recruited macrophages in ischemic muscles of hyperlipidemic and type 2 diabetes mellitus mice revealed that* Cfb*,* Serping*, and* Tnfsf15* promoters were hypomethylated, whilst* Arg1*,* Nrp1*,* Cxcr4*,* Plxnd1*,* Cdk18,* and* Fes* were significantly hypermethylated in inflamed tissues, skewing macrophage phenotype to the M1 lineage [39].

Similarly, DNMT3B is activated in adipose tissue macrophages of obese mice and silences the M2 TF PPAR-*γ* via methylation of CpG sites on its promoter [42]. Additional supporting evidence comes from atherosclerosis studies, where inhibition of DNA methyltransferases in macrophages results in a severe reduction in migration to plaques, adhesion to the endothelium, and secretion of a broad range of proinflammatory cytokines, chemokines, and adhesion molecules [105]. LXR*α* and PPAR-*γ* CpG sites were shown to be hypomethylated providing a possible explanation for the anti-inflammatory phenotype of macrophages.

## 6. Histone Demethylases (DMTs)

In contrast to HMTs, DMTs enzymes remove methyl groups from histones exhibiting dynamics of chromatin remodeling and constant regulation of gene expression. There are seven subfamilies of DMTs with high specificity for their substrates (Table 2). H3K4 mono- and dimethylation are removed by KDM1 members, but KDM2, KDM5, and KDM6 members can also demethylate H3K4 [14]. H3K9 mono- and dimethylation are reversed by KDM3 and KDM7 subfamily members, whereas KDM4 were the first proteins identified to catalyse removal of di- and trimethylation from histones. The negative marks on H3K36 tails are removed by KDM2 (mono- and dimethylation) and KDM4 (di- and trimethylation). Finally, di- and trimethylation on H3K27 are demethylated by KDM6, whereas mono- and dimethylation on H3K27 are removed by KDM7 [14].

DMTs modulate polarisation of macrophages to both M1 and M2 states (Figure 2(a)). The KDM1 member AOF1 was shown to be recruited by c-Rel to* Il12b*,* Mdc*, and* Ip10* promoters in DCs and macrophages stimulated with LPS [43]. Recruited AOF1 demethylates H3K9me2 and is involved in a feed-forward circuit to attract more NF-kB molecules and initiate the transcription of proinflammatory genes [43]. Similarly, the KDM4 family member JMJD2D was reported to attack H3K9me3 levels around the enhancers of* Il12b* and* Mdc* genes in DCs and macrophages upon stimulation with LPS and release them from active repression [44].

The most widely studied demethylase in the macrophage polarisation field is the KDM6 member, JMJD3. JMJD3 has been reported to affect multiple cellular processes under inflammatory conditions in macrophages, such as transcription of inflammatory genes, oxidative stress, chromatin remodelling, cell proliferation, and differentiation [106]. JMJD3 expression is rapidly upregulated in LPS-activated microglia through existing NF-kB molecules, STAT1/STAT3 [107, 108] and together with the other KDM6 member, UTX, it contributes to the establishment of the M1 phenotype by tuning gene transcription early on after stimulation [109], before removing methyl groups from H3K27me3-repressed M1 signature genes, such as* TNF* [45]. JMJD3's role was demonstrated in an* in silico* study, where it was predicted to target the CD40, chemokine, and IFN signalling pathways [46]. Another line of evidence suggests that in human type 2 diabetes nonhealing wounds and diet-induced obese mice, JMJD3 is responsible for the elevated IL-12/IL-10 ratio [47] and it has also been reported to be expressed in serum amyloid A-primed murine macrophages as part of the proinflammatory cytokine secretion program [48].

In contrast, other reports have shown that deficiency of JMJD3 in microglia leads to enhanced proinflammatory mediator secretion, such as TNF-*α* and IL-6, and a reduction in the M2 markers, Arginase-1, and CD206, creating a hostile microenvironment for neurons [49]. This result suggests that JMJD3 may also be induced by alternative activation. Indeed, JMJD3 is expressed in IL-4-stimulated BMDM as a direct downstream target of STAT6 [50] and promotes the expression of* Irf4* to establish a M2 phenotype [51].

Recently, a novel family of Fe^2+^- and 2-oxoglutarate-dependent dioxygenases [110], named ten-eleven translocation (TET) proteins (reviewed in [111]), were found to take part in a number of biological processes, such as embryonic development [112] and epigenetic regulation of gene transcription and cancer [113], and mediate their effects by oxidising 5-methylcytosine in DNA to 5-hydroxymethylcytosine, 5-formylcytosine, and 5-carboxylcytosine [114, 115].

Although TET2 and TET3 are expressed in macrophages, their levels are not increased upon LPS stimulation, implying that they may not play a critical role in DNA demethylation during macrophage activation [116]. However, Zhang et al. showed that TET2 actively represses IL-6 during the resolution phase of inflammation [52]. The authors found that IkB*ζ* targets TET2 to the* Il6* promoter to indirectly recruit HDAC2, which deacetylates H3 and H4 histones and suppresses transcription.

Therefore, TET enzymes warrant further investigation as they may regulate macrophage polarisation either directly through conversion of cytosines in gene promoter DNA sequences or indirectly via recruitment of histone modifying enzymes.

## 7. Histone Acetyltransferases (HATs)

Histone acetyltransferases are enzymes that transfer acetyl groups to core histones, which has subsequent effects on chromatin remodelling and gene expression. They are a diverse group of proteins with several catalytic domains that dictate their subunit specificity and functions. HATs are divided into two families [117]: Gcn5 N-acetyltransferases (GNATs) and Morf, Ybf2, Sas2, and Tip60 HATs. Other proteins, such as p300/CBP, Taf1, and nuclear receptor coactivators also possess catalytic acetyltransferase activities, but no typical HATs domains. These proteins are considered as an orphan class of HATs [117].

HATs have been shown to be involved in initiating gene expression in macrophages during inflammation (Table 3). However, to date only a limited number of reports have detailed how HATs catalyse the expression of specific M1 or M2 associated genes. Instead, we only have a more global understanding of histone acetylation and its role in regulating gene expression.

Soluble/secreted factors from the parasite* M. corti* were shown to downregulate* Tnf*,* Il6*,* Nos2*,* H2-Eb1*, and* Ciita* expression in LPS-primed microglia. These factors suppressed H3K4me3 and H3K9/14Ac in these genes and promoted RNA polymerase II recruitment to the* Arg1* promoter, causing compromised immune responses of microglia in murine neurocysticercosis [53].

HATs may also interact with the opposing histone deacetylase enzymes to enhance acetylation and eventually activation of antiviral gene promoters. For example, p300/CBP was shown to be recruited to the inactive* Ifnα* promoter upon IRF5 phosphorylation and displace the SMRT/Sin3a repressive complexes. IRF5 is subsequently acetylated by p300/CBP facilitating H3 histone acetylation of target genes, including* Tnf* and* Il6* [54].

## 8. Histone Deacetylases (HDACs)

The enzymes that oppose HATs functions are referred to as HDACs. Histone deacetylation is a dynamic process and may be the result of other posttranslational modifications. HDACs functions may induce further epigenetic changes and alternative gene expression (Table 4). To date there have been eighteen identified mammalian HDACs, which are classified in five groups: Class I (HDAC1, HDAC2, HDAC3, and HDAC8), Class IIa (HDAC4, HDAC5, HDAC7, and HDAC9), Class IIb (HDAC6 and HDAC10), and Class III (consists of the NAD^+^-dependent HDACs) and HDAC11, which constitutes a class of its own [118].

HDACs effects during macrophage activation (Figure 2(b)) have primarily been studied through the use of small molecule inhibitors [119]. For example, inhibition of histone deacetylases with the HDACs classes I and II inhibitor Trichostatin A (TSA) in differentiating bone marrow cells arrests cells to the phase of proliferating progenitors. [60]. Furthermore, simultaneous inhibition of HDACs classes I and II in several macrophage populations results in reduced levels of pattern recognition receptors, activation markers, cytokines, and chemokines. Secretion of reactive oxygen species, NO, and modulation of cellular processes, such as chemotaxis, phagocytosis, apoptosis, and cellular metabolism, have also been reported to be affected [30, 55–57]. In a study by Lugrin et al., the authors showed that the proinflammatory mediator macrophage inhibitory factor (MIF) is a downstream target of HDACs inhibition [58]. Inhibition of HDACs may also be beneficial in a complex inflammatory environment, whereby the interactions of macrophage populations with other resident cells are detrimental to the host. In this respect, HDACs inhibition rescued oligodendrocytes during traumatic brain injury via induction of the M2 phenotype in resident microglia [59].

There are many plausible explanations for the HDACs class I and II effects on macrophage activation status. Roger et al. reported that HDACs inhibition enhances the recruitment of the repressive complex Mi-2b to the promoters of M1 activation state genes, such as* Il6* [55]. Another possibility is that these effects are a result of the decline in the PU.1 levels in macrophages treated with TSA [60, 61]. Interestingly, Serrat et al. proposed that TSA induces an acetylation-mediated repression on C/EBP*β*, which binds with lower efficiency to the* Arg1* promoter in macrophages [62].

Use of a selective class I HDACs inhibitor, valproic acid (VPA), has been shown to reduce expression of M1-associated genes in macrophages, including CD40, CD80, and proinflammatory cytokines [63], implying that the members of this subfamily promote M1 activation in macrophages. Indeed, HDAC1, HDAC2, and HDAC3 were shown to act as a network of enzymes, aiming to enhance LPS-responsiveness in macrophages. Strikingly, this effect is mediated not only by histone tail modulation, but also via nonhistone protein phosphorylation and acetylation [120, 121].

Class I HDAC1 releases the* IFNA* promoter from bound repressive complexes upon interaction with p300/CBP and subsequent acetylation and transcription initiation [54]. Halili et al. expanded our understanding of this enzyme's actions. The authors reported that a HDAC1 inhibitor increased the expression of* Cox2* and* Pai1* and reduced* Edn1*, indicating that HDAC1 may have a protective role in inflammatory diseases [64]. HDAC2 was reported to deacetylate and therefore modulate* Ciita* expression in macrophages [65]. In atherosclerotic plaques, such an effect may prove to be protective; CIITA promotes expression of MHC-II and antigen presentation to T cells, a pivotal step in transition to chronic inflammation, whilst inhibition of CIITA rescues collagen deposition by smooth muscle cells and eliminates plaque vulnerability.

Studies of HDAC3 demonstrate that it promotes macrophage responsiveness to LPS via IFN-*β* production [66]. This deacetylase can lead to enhanced IL-6 and NO secretion and inhibition of TGF-*β* in an atherosclerosis model [30, 67], whereas HDAC3's proinflammatory effects in macrophages reflect its ability to bind to PU.1 and inhibit H3K9 acetylation in M2 signature genes [122].

Class IIa HDACs have also been shown to promote an M1 phenotype. In particular, a HDAC7 isoform that lacks the N-terminal 22 amino acids was reported to interact with the newly transcribed hypoxia-inducible factor 1-alpha and induce the expression of* Il12b*,* Il6*, and* Edn1* in TLR4-stimulated macrophages [68]. Another member, HDAC9, is associated with disease progression in LDLR^−/−^ mice. Deletion of this histone modifier resulted in improved levels of HDL and LDL due to ABCA1 and ABCG1 upregulation and macrophage polarisation to the M2 phenotype [69]. In a recent report, HDAC6 inhibitors were shown to induce the expression of* Edn1* and* Il12b* [64]. However, the authors concluded that HDAC6 might not work alone as HDAC6^−/−^ BMDM showed normal LPS-induced expression of HDACs-dependent genes.

Studies with the Class III HDACs have revealed that members of this enzyme subfamily behave diversely. SIRT1 levels in macrophages are dampened under inflammatory conditions, suggesting that this HDACs and the epigenetic changes it is responsible for are not required for cell activation [71]. Indeed, studies have shown that SIRT1 deficiency in myeloid cells results in increased tissue infiltration of M1 macrophages and augments inflammatory responses, mainly due to NF-kB p65, AP-1, and FAK increased acetylation and increased target gene expression [70, 72–74]. In ApoE^−/−^ mice, haploinsufficiency of SIRT1 led to augmented macrophage oxLDL uptake and increased foam cell formation [123].

Deficiency of another member of the Sirtuin family, SIRT2, exacerbated DSS colitis in mice, exemplified by higher TNF-*α* and IL-1*β* levels, and impaired epithelial integrity [75]. Subsequent reports confirmed that SIRT2 deficiency increased the expression of proinflammatory genes, reactive oxygen and nitrite species, and activation surface markers in microglia and macrophage cell lines [76, 77]. Interestingly, SIRT2 has been shown to act as an NF-kB p65 deacetylase, placing microglia inflammatory responses under control. Phosphorylation on serine 331 inactivates SIRT2 and allows cells to undergo a proinflammatory cycle, which ultimately leads to death and neurotoxicity in the CNS [77].

Sirtuins can form a complex network of enzymes acting in concert to regulate inflammatory responses initiated by macrophages. Strikingly, SIRT6 was shown to compensate for the loss of SIRT1 in macrophages by controlling the* IkB* promoter acetylation status and subsequent* Il1β* expression [72].

The functions of the only HDACs Class IV member are only now being unravelled. A couple of reports found that HDAC11 regulates the IL-12/IL-10 ratio in antigen presenting cells [78, 79]. HDAC11 was found to bind to the proximal site of the IL-10 promoter and modulate the recruitment of PU.1, Sp1, and STAT3 at late stages of LPS activation. Given the pivotal role of IL-12 and IL-10 in T cell activation and tolerance, this enzyme holds a lot of promise in therapeutic intervention, where manipulation of the adaptive immune response is of critical importance [79]. Another role attributed to HDAC11 was IL-1*β* suppression in DCs and macrophages during LPS stimulation [80]. In fact, HDACs inhibition led to upregulation of IL-1*β* cleavage and maturation in a caspase 8-dependent manner, demonstrating that HDACs inhibition may prove to be more challenging than originally thought.

## 9. Concluding Remarks

Research in the field of epigenetic regulation of macrophages during inflammation has flourished in the last decade. We now know that methylation and acetylation sites on core histones adjacent to inflammation-related genes are heavily affected by epigenetic enzymes, which contribute to the establishment and maintenance of M1 or M2 phenotypes and therefore dictate the magnitude and type of immune response mounted.

HMTs are strongly associated with M2 activation by repressing M1 phenotype signature genes and promoting the transcription of M2 genes. Repression may occur in the basal state or during inflammation. In contrast, DMTs have been linked with the M1 phenotype as a result of demethylation of repressive sites on histones. These findings imply that although demethylation of core histones can be mainly linked to derepression of genes, methylation can be associated with both gene expression when it is positive (e.g., H3K4) or inhibition when negative (e.g., H3K9 and H3K27). However, the timing of histone methylation/demethylation in close proximity to gene promoters is crucial.

Accumulation of the negative H3K9me2 motif at primary response gene promoters is sufficient to actively suppress transcription initiation [32]. Whether HMTs are recruited to gene promoters as a result of corepressor complexes is a common question that has been posed in recent reports and suggests that the level of gene regulation is complex and may outweigh a model of random attraction of these enzymes to gene promoters [38].

In fact, negative regulation of gene expression may involve networks of HMTs and corepressor complexes that work cooperatively to control the derepression of a cluster of primary response genes. In accordance with this, a different level of cell-specific and stimulus-induced gene repression in DCs and macrophages was shown to be achieved by deposition of H3K9me3 at upstream broad enhancers of inflammation-related genes [44]. This method of gene repression seems to confer cell type-specific protection from excessive secretion of proinflammatory mediators in the basal state and the transcriptional constraints can be applied to various genes simultaneously [44].

As a generic model, it seems plausible to hypothesise that HMTs need guidance by a “molecular beacon” at the promoter DNA sequence in order to bind and methylate their substrates. This can be mediated by corepressor complexes, such as NcoR, SMRT, and mSin3A, as described earlier, that provide the anchor sites for the HMTs adjacent to gene promoters and enhancers. Further investigation into the patterns of interactions between corepressor complexes and HMTs is needed and might reveal that gene regulation in macrophages in the basal state is not as random as it may seem and might be heavily dependent on the corepressor/activator complex balance at gene promoters.

Proinflammatory mediator expression during inflammation may be downregulated either by negative methylation effects of recruited HMTs at gene promoters or indirectly via positive methylation (and transcription initiation) of M2 signature or anti-inflammatory genes [36]. Hence, the assessment of HMTs and DMTs in macrophage polarisation needs to take into account indirect effects of these enzymes on genes, such as TFs that actively bind to the target gene promoters and drive the response to the M1 or the M2 phenotype.

To conclude, the epigenetic changes that occur in macrophages during activation by various environmental stimuli have attracted much interest. The strategic location of macrophage populations complemented by the ability to shape their functions according to the needs of the host makes the enzymes responsible for their polarisation, a potential target for therapeutic interventions. Although there are still many open questions regarding the mode of action and the interactions of these enzymes, the knowledge we have acquired over the last decade hints for more effort to understand the molecular pathways involved in the regulation of gene expression of macrophages during inflammation and the design of therapies to tackle acute and chronic inflammatory diseases.

## Figures and Tables

**Table 1:** HMTs and DNMT involved in macrophage polarisation.

Name	Family	Function	References
HMTs	—	IL-6, IL-12, TNF-*α*, NO secretion induction, and IL-1*β* secretion inhibition	[30]
SETDB1	KMT1	*TNF* repression	[31]
EHMT2	KMT1	*Ifnβ* and ISG suppression	[32]
EHMT1, EHMT2	KMT1	M2-IL-4 activation status	[33]
MLL	KMT2	*CXCL10* induction	[34]
MLL4	KMT2	CD14 membrane anchoring	[35]
Ash1	KMT2	A20 production and IL-6 suppression	[36]
SETD1A, ASH1, MLL3, and MLL4	KMT2	M2-IL-4 activation status	[33]
SMYD2	KMT3	*Tnf*, *Il6*, MHC-II, and CD40/80 suppression	[37]
SMYD3	KMT3	*Alox15* induction	[34]
SMYD5	KMT3	*Il1α*,* Il1β*,* Ccl4*, *Tnf*, and *Cxcl10* repression	[38]
SUV420H2 and SETD8	KMT5	M2-IL-4 activation status	[33]
EZH1	KMT6	M2-IL-4 activation status	[33]
DNMT	—	*Cfb*, *Serping*, *Tnfsf15* induction *Arg1*, *Nrp1*, *Cxcr4*, *Plxnd1*, *Cdk18*, and *Fes *repression	[39]
DNMT1	—	*Socs1 *silencing	[40]
DNMT1 and DNMT3b	—	Cystathionine-*γ*-lyase suppression	[41]
DNMT3B	—	PPAR-*γ* silencing and polarisation to M1 phenotype	[42]
DNMT3A and DNMT3L	—	M2-IL-4 activation status	[33]

**Table 2:** DMTs involved in macrophage polarisation.

Name	Family	Function	References
AOF1	KDM1	*Mdc*, *Il12b*, and *Ip10* induction	[43]
KDM2A	KDM2	M2-IL-4 activation status	[33]
JMJD2D	KDM4	*Il12b* and *Mdc* induction	[44]
JMJD3/UTX	KDM6	TNF-*α* secretion	[45]
JMJD3	KDM6	NF-kB, CD40, and IFN signalling	[46]
JMJD3	KDM6	IL-12/IL-10 increase	[47]
JMJD3	KDM6	*Il12b*, *Il23a*, *G-CSF*, and *Trem1* induction	[48]
JMJD3	KDM6	Arginase-1, *CD206* induction, TNF-*α*, and IL-6 repression	[49]
JMJD3	KDM6	*Arg1*, *Retnla*, and *Chi3l3* induction	[50]
JMJD3	KDM6	*Arg1*,* Fizz1, Ym1*, *Mrc1*, and *Il13 *induction	[51]
JMJD3	KDM6	*Irf4 *induction	[49, 51]
PHF2	KDM7	*Tnf*, *Ccl4*, *Cxcl10, Il1a*,* Il1b*, *Il6*,* Ccl5*,* Irf1*, Mx1, and Oas3 induction	[38]
TET2	TET	*Il6 *repression	[52]

**Table 3:** HATs involved in macrophage polarisation.

Target	Function	References
H3K9/14	TNF-*α*, IL-6, NOS2, MHC-II, and CIITA induction	[53]
H3	*IFNA*, *TNF*, and *IL6* expression	[54]

**Table 4:** HDACs involved in macrophage polarisation.

Name	Class	Function	References
—	Classes I and II	Pattern recognition receptors, activation markers, cytokines, chemokines, secretion of reactive oxygen species, and NO induction	[30, 55–57]
—	Classes I and II	MIF induction	[58]
—	Classes I and II	*Cd16* induction, *Cd206*, TNF-*α*, and NO repression	[59]
—	Classes I and II	PU.1 induction	[60, 61]
—	Classes I and II	*Arg1* induction	[62]
—	Class I	IL-12, TNF-*α* secretion, IL-10 repression, and CD40/80 induction	[63]
HDAC1	Class I	*IFNA* and* IL6* repression	[54]
HDAC1	Class I	*Cox2*, *Pai1* inhibition, and *Edn1* induction	[64]
HDAC2	Class I	*Ciita* inhibition	[65]
HDAC2	Class I	M2-IL4 activation status	[33]
HDAC2	Class I	*Il6* repression	[52]
HDAC3	Class I	IFN-*β* secretion	[66]
HDAC3	Class I	TGF-*β* suppression	[67]
HDAC3	Class I	IL-6, NO secretion	[30]
HDAC6	Class IIb	*Edn1* and *Il12b* induction	[64]
HDAC7	Class IIa	*Il12b*, *Il6*, and *Edn1* induction	[68]
HDAC9	Class IIa	PPAR-*γ*, ABCA1, and ABCG1 repression	[69]
HDAC9	Class IIa	M2-IL4 activation status	[33]
SIRT1	Class III	*Tnf*, *Il6*, *Nos2*, and *Mcp1* represion	[70]
SIRT1	Class III	TNF-*α*, IL-1*β*, IL-6, IL-12, and MCP-1 inhibition	[71–73]
SIRT1	Class III	PGE_2_ inhibition	[74]
SIRT1	Class III	M2-IL4 activation status	[33]
SIRT2	Class III	*Tnf*, *Il6*, *Mcp1* repression, *Il4r*, and *Il10* induction	[75]
SIRT2	Class III	*Tnf*,* Il1β*,* Il6*, *Cox2*,* Nos2*, and reactive oxygen species inhibition	[76]
SIRT2	Class III	iNOS, TNF-*α*, IL-6, CD40/80, IL-10, and reactive oxygen species inhibition	[77]
SIRT6	Class III	*Il1β* repression	[72]
HDAC11	Class IV	*Il10 *downregulation	[78, 79]
HDAC11	Class IV	IL-1*β* repression	[80]

## Abbreviations

AA: Arachidonic acid
DAMPs: Danger-associated molecular patterns
DCs: Dendritic cells
DNMT: DNA methyltransferase
DMTs: Histone demethylases
ES cell: Embryonic stem cell
GNATs: Gcn5 N-acetyltransferases
HATs: Histone acetyltransferases
HDACs: Histone deacetylases
HETE: Hydroxyeicosatetraenoic acid
HMTs: Histone methyltransferases
ISREs: Interferon-sensitive response elements
ISG: Interferon-stimulated gene
MIF: Macrophage inhibitory factor
PAMPs: Pathogen-associated molecular patterns
SET: Suppressor of variegation-Enhancer of zeste-Trithorax
TET: Ten-eleven translocation
TF: Transcription factor
TSA: Trichostatin A
VPA: Valproic acid.
